# Cholera

**Published:** 1853-09

**Authors:** 


### CHOLERA.

Cholera was reported to be prevalent in Cumberland, Md., some time since, but a few days ago we saw a statement that a number of the physicians of that town denied that the disease was cholera. Our impression is that this will turn out to be the fact. Certainly the complaint is not prevalent in any other part of our country, and our belief is that it as dissappeared from the united States for the present.

